# Development of High Resolution Melting Analysis for the Diagnosis of Human Malaria

**DOI:** 10.1038/srep15671

**Published:** 2015-10-28

**Authors:** Kek Heng Chua, Siew Chee Lim, Ching Ching Ng, Ping Chin Lee, Yvonne Ai Lian Lim, Tze Pheng Lau, Hwa Chia Chai

**Affiliations:** 1 Department of Biomedical Science, Faculty of Medicine, University of Malaya, 50603 Kuala Lumpur, Malaysia; 2 Institutue of Biological Sciences, Faculty of Science, University of Malaya, 50603 Kuala Lumpur, Malaysia; 3 School of Science and Technology, Universiti Malaysia Sabah, Jalan UMS, 88400, Kota Kinabalu, Sabah, Malaysia; 4 Department of Parasitology, Faculty of Medicine, University of Malaya, 50603 Kuala Lumpur, Malaysia; 5 Department of Pathology, Faculty of Medicine, University of Malaya, 50603 Kuala Lumpur, Malaysia

## Abstract

Molecular detection has overcome limitations of microscopic examination by providing greater sensitivity and specificity in *Plasmodium* species detection. The objective of the present study was to develop a quantitative real-time polymerase chain reaction coupled with high-resolution melting (qRT-PCR-HRM) assay for rapid, accurate and simultaneous detection of all five human *Plasmodium* spp. A pair of primers targeted the 18S SSU rRNA gene of the *Plasmodium* spp. was designed for qRT-PCR-HRM assay development. Analytical sensitivity and specificity of the assay were evaluated. Samples collected from 229 malaria suspected patients recruited from Sabah, Malaysia were screened using the assay and results were compared with data obtained using PlasmoNex^TM^, a hexaplex PCR system. The qRT-PCR-HRM assay was able to detect and discriminate the five *Plasmodium* spp. with lowest detection limits of 1–100 copy numbers without nonspecific amplifications. The detection of *Plasmodium* spp. in clinical samples using this assay also achieved 100% concordance with that obtained using PlasmoNex^TM^. This indicated that the diagnostic sensitivity and specificity of this assay in *Plasmodium* spp. detection is comparable with those of PlasmoNex^TM^. The qRT-PCR-HRM assay is simple, produces results in two hours and enables high-throughput screening. Thus, it is an alternative method for rapid and accurate malaria diagnosis.

Symptoms of malaria are nonspecific and similar to other diseases. Without prompt and proper treatment, mild and moderate malaria may also be lethal in children and some adult patients especially for those infected with *Plasmodium falciparum*. Cases with *P. falciparum* have higher rates of complications and the symptoms of malaria may suddenly develop, possibly leading to death within 24 hours if treatment is not provided^1^,^2^. Hence, a few main factors need to be taken into account when developing a malaria diagnostic kit: rapidity, accuracy, sensitivity, cost-effectiveness, easy handling, and minimal training of personnel^3^.

Microscopic examination of blood films remains as the most common malaria diagnostic method due to its inexpensiveness and simplicity. It is considered as the “gold standard” for malaria diagnosis^4^. However, since various limitations which include the requirement of skilled personnel, low sensitivity (100 to 200 parasites/μl of blood), time-consuming and unreliability in species identification especially of *P. knowlesi* due to its similarities with *P. falciparum* in the early ring form stage and *P. malariae* in the latter stages, the microscopic technique needs to be coupled with other alternative diagnostic methods to heighten the accuracy of the identification^4^,^5^. Available complementary methods include rapid diagnostic tests (RDTs) and polymerase chain reaction (PCR)-based techniques.

Since the late 1980s, polymerase chain reaction (PCR)-based methods have been applied in malaria diagnosis due to their high sensitivity, rapidity and reproducibility^6^,^7^,^8^. Molecular detection using conventional nested PCR techniques offers detection of parasites at lower concentration of 5 parasites/μl^9^,^10^ and mixed infections, as well as discrimination of the *Plasmodium* spp^6^,^11^,^12^. A number of nested and semi-nested PCR methods have been developed over the years^6^,^7^,^11^,^13^,^14^,^15^,^16^, which allow for detection of up to four human *Plasmodium* spp. (*P. falciparum, P. vivax, P. malariae* and *P. ovale*), and these methods were replicated in other studies and epidemiological surveillance of malaria cases in numerous countries^14^,^15^,^17^,^18^,^19^. When simple dried blood spot sampling on filter papers is coupled with boiling method for DNA extraction, this has increased the robustness of nested PCR Malaria detection system^20^,^21^,^22^. Later on, single round multiplex PCR methods were also developed to overcome the disadvantages of nested and semi-nested PCR methods, such as time-consuming and risk of contamination^23^,^24^,^25^,^26^. PlasmoNex^TM^ (Reszon Diagnostic International, Selangor, Malaysia) is the commercially available malaria diagnostic kit developed from our previous study which enables detection of the five currently most important human *Plasmodium* spp. (including *P. knowlesi*) using hexaplex PCR system^26^. The emergence of quantitative real-time PCR (qRT-PCR) technology has brought the malaria diagnosis to another level, with greater sensitivity (down to 0.02 parasite/μl)^27^, easier execution and no post-PCR manipulations compared to the conventional PCR methods. There are two types of qRT-PCR developed for malaria diagnosis: i) fluorescent dye-based qRT-PCR with the use of nonspecific double-stranded DNA (dsDNA) intercalating dyes such as SYBR green^28^,^29^,^30^,^31^, and ii) probe-based qRT-PCR with the use of specific fluorescent probes such as TaqMan probes^32^,^33^,^34^,^35^,^36^,^37^,^38^,^39^. In general, qRT-PCR assay using dsDNA intercalating dye requires lower running cost than that using fluorescent probes. However, intercalating dyes will bind to any dsDNA including non-specific dsDNA sequences and tend to generate false positive results. In 2004, a ready-to-used qRT-PCR system, namely Real Art Malaria LC PCR assay, was introduced and made commercially available in the market. However, Farcas and colleagues^35^ evaluated this commercial assay on febrile returned travellers and found that it failed to differentiate four *Plasmodium* spp.

qRT-PCR-SYBR green assay which can detect quantitatively up to five species of *Plasmodium* has been developed by Oddoux and colleagues^31^. However, they used a total of nine primers to amplify *P. falciparum*, *P. vivax*, *P. malariae*, *P. ovale* and *P. knowlesi*. When the number of primers increases, the risk of unspecific binding between primers also increases. In the present study, we developed a quantitative real-time PCR method for the detection of the five human-infecting *Plasmodium* spp., but instead of SYBR green dye, high resolution melting (HRM) analysis was incorporated. Similar to qRT-PCR-SYBR green technique, the steps in HRM analysis involve amplification of the region of interest in the presence of a specialized dsDNA binding dye and gradual denaturation of amplicons by increasing the temperature in small increments in order to produce a characteristic melting profile that is called melting analysis. The SYTO^®^ 9 dye used in the HRM analysis produces highly reproducible DNA melting curves over a broader range of dye concentrations than does SYBR Green I^40^. It is also far less toxic than SYBR Green I and does not appear to selectively detect particular amplicons^40^. Hence, SYTO^®^ 9 can be used at higher concentration than SYBR Green I for greater saturation of the dsDNA sample in the assay, reducing dye redistribution to non-denatured regions during dissociation of dsDNA^41^. Owing to this characteristic, fluorescent signals measured in the assay have higher fidelity and provides greater resolution to melting curve analysis. This method has been employed and coupled with nested PCR technique for human malaria diagnostic by Kipanga and colleagues^42^, with limit of detection of 0.236 parasites/μl. However, it required two rounds of PCR and was able to detect only three species of *Plasmodium*. Our study aimed to design single-round of qPCR-HRM assay which used lower number of primers to detect all five species of human plasmodium with increased specificity and sensitivity.

## Methods

### Plasmid DNA preparation

All plasmids were obtained from previous study^26^. The plasmids containing the conserved region of 18S SSU rRNA gene sequence for each *Plasmodium* spp. were constructed and served as positive controls to assess the sensitivity and specificity of qRT-PCR-HRM assay.

*Escherichia coli* carrying the desired recombinant plasmid DNA was grown overnight in 10 ml Luria Bertani (LB) broth (BD, NJ, USA) containing 100 μg/ml ampicillin (Merck, Darmstadt, Germany) at 37 °C with vigorous shaking. The bacterial culture was harvested by centrifugation at 6000 × g for 15 minutes at 4 °C. The supernatant were removed and the cell pellets consisting of the recombinant plasmids of the five *Plasmodium* spp. were isolated and purified with High Yield Plasmid Mini Kit (Yeastern Biotech, Taiwan) according to the manufacturer’s instructions. The purified plasmid DNA samples were quantified spectrophotometrically at 260 nm and then kept at −20 °C.

### Clinical samples

A total of 229 clinical DNA samples were collected from Sabah, Malaysia from 2008 to 2010. Prior to sample collection, approval of the ethics application with appropriate experimental protocols was obtained from the Medical Ethics Committee of University of Malaya Medical Centre, Kuala Lumpur, Malaysia. All the protocols used in this study were in accordance with the approved guidelines (Ethics reference no. 709.2) and informed consents were obtained from all patients recruited. Peripheral blood was drawn into EDTA tube (BD) from each patient suspected with malaria but prior to anti-malarial treatment, and an aliquot of each blood sample was subject to routine biological diagnosis. Two hundred μl of the remaining blood sample was extracted according to the manufacturer’s instruction to obtain total human genomic DNA as well as plasmodial DNA using QIAamp DNA mini kit (Qiagen, Hilden, Germany). All the samples were initially assessed using PlasmoNex^TM^ (Reszon Diagnostic International), a hexaplex PCR detection system^30^. From the results, the clinical samples were found to consist of 92 cases of *P. falciparum* cases, 1 case of *P. ovale*, 2 cases of *P. malariae* cases, 90 cases of *P. vivax* cases, 36 cases of *P. knowlesi* cases, 2 cases of mixed infections (*P. falciparum* and *P. vivax*), and 6 cases with negative results.

### Microscopic examination

Microscopic examination for clinical samples was done in previous study^26^. The analysis was performed by experienced microscopists in hospital, and the parasitemia level was estimated. Briefly, thin and thick blood smears were prepared and examined under microscopic magnification of ×1000 for the presence of *Plasmodium*. The parasite counts were estimated with scores ranging from + to ++++, whereby + indicated 4 to 40 parasites/μl, ++ indicated 41 to 400 parasites/μl, +++ indicated 401 to 4000 parasites/μl, and ++++ indicated >4000 parasites/μl of blood^43^. Parasitemia level was defined as negative if blood films was absence of parasites in 300 microscopic fields.

### Quantitative real-time PCR-HRM (qRT-PCR-HRM) assay development

#### Primer design

Primers were designed from 18S SSU rRNA gene of the five *Plasmodium* spp. as it contains both highly conserved and variable regions. The 18S SSU rRNA gene sequences of *P. falciparum*, *P. knowlesi*, *P. ovale*, *P. malariae* and *P. vivax* were obtained from GenBank^43^ (NCBI, BethesdaMD, USA) (accession number M19172.1, U83876.1, L48987.1, M54897.1 and X13926.1, respectively) and sequence alignment was carried out using BioEdit Sequence Alignment Editor v7.1.7^44^. Once the specific and conserved regions for each *Plasmodium* spp. were identified, a pair of common primers was designed for the qRT-PCR-HRM assay using Primer 3 software^45^, a free online primer design tool (http://simgene.com/Primer3). BLAST analysis (NCBI) was performed on the primer pair to evaluate their specificity. The folding characteristics of these primers and amplicons were evaluated using an internet open access secondary structure profiling software, OligoAnalyzer 3.1 (http://sg.idtdna.com/calc/analyzer) (Integrated DNA Technologies, Coralville, Iowa, USA).

### Evaluation of primer efficiency with various annealing temperatures

A series of conventional gradient PCR was carried out to make sure the primer pair was able to amplify the target region of all the five *Plasmodium* spp. without producing unspecific bands or primer dimers which would interfere with the result interpretation in qRT-PCR-HRM analysis later on. Overall, 20 μl of PCR mixture was prepared, which contained 1× *Taq* buffer, 1.5 mM of MgCl_2_, 0.2 mM of dNTPs, 0.5 μM of each primer, 1 U of *Taq* polymerase and 10 ng of DNA template (plasmid carrying target sequence of each *Plasmodium* spp.). Conventional gradient PCR was performed using Applied Biosystems Veriti^®^ 96 well thermal cycler (Applied Biosystems, CA, USA). The mixture was heated at 95 °C for 1 minute, followed by 30 cycles of denaturation at 95 °C for 30 seconds, annealing at gradient temperatures from 59 °C to 64 °C for 30 seconds and extension at 72 °C for 30 seconds, and final elongation at 72 °C for 5 minutes. After the gradient conventional PCR, the PCR products were electrophoresed and analysed on 2% (w/v) agarose gel.

### Performing qRT-PCR-HRM analysis

qRT-PCR-HRM assay was performed using an Applied Biosystems 7500 Fast Real-Time PCR System. Each reaction was carried out in a total volume of 20 μl reaction mixture containing 10 μl of 2× MeltDoctor^®^ HRM master mix (Applied Biosystems), 0.1 μM of each primer and 1 μl of 10 ng plasmid DNA bearing target sequence of each *Plasmodium* spp. or 1 μl of each clinical DNA sample. Both MicroAmp^®^ Fast 0.1 ml 8-tube strip with cap (Applied Biosystems) and MicroAmp^®^ Fast Optical 0.1 ml 96-well reaction plate (Applied Biosystems), sealed with MicroAmp^®^ Optical adhesive film (Applied Biosystems), were used to perform qRT-PCR-HRM. The tubes or plates were centrifuged briefly prior to undergoing qRT-PCR-HRM.

The thermal profile comprised initialization step at 95 °C for 10 minutes, followed by 40 cycles of amplification which included denaturation of double stranded DNA at 95 °C for 15 seconds and annealing of DNA strands at 60 °C for 1 minute. The PCR products were then subject to a melt program: denaturation of double stranded DNA at 95 °C for 10 seconds, annealing of double stranded DNA at 60 °C for 1 minute, followed by a gradual temperature increase until 95 °C in 15 seconds and annealing of DNA at 60 °C for 15 seconds. Finally, melting curve plots were generated and analysed using High Resolution Melt software v 3.0.1 (Applied Biosystems) to determine average melting temperature (T_m_) for each *Plasmodium* spp.

### Analytical sensitivity and specificity evaluation

The analytical sensitivity or detection limit of qRT-PCR-HRM assay was assessed by using 10-fold serial dilutions of positive control plasmid DNAs, ranging from 100,000 to 1 copy number(s)/μl. The procedure was repeated twice to ensure the reproducibility of the threshold cycle number (Ct) results. Standard curves were also plotted from Ct’s of the 10-fold serial diluted plasmid DNAs using 7500 Software v2.0.1 (Applied Biosystems) to determine the efficiency percentage of each *Plasmodium* spp. assay.

The specificity of the assay was also evaluated using DNA of other organisms such as human genomic DNA; bacterial DNA of *Aeromonas hydrophila, A. punctata, Vibrio parahaemolyticus, V. harveyi*; and parasitic DNA of *Toxoplasma gondii, Ancylostoma ceylanicum, Haemonchus contortus, Trichostrongylus colubriformis, Nippostrongylus brasiliensis, Giardia duodenalis* and *Entamoeba histolytica*. The assay was performed in duplicates with 10 ng of each DNA.

### Testing for detection of mixed infections

Mock mixed infections were created by spiking plasmid DNA carrying target sequences from two different species at varying ratios [1–10,000 copy number(s)/species] into 50 ng/μl of human DNA sample. Mock samples were subject to qRT-PCR-HRM and the ability of the assay to detect the species present in mock samples was examined. Mock samples with three, four and five mixed *Plasmodium* species (10,000 copy numbers/species) were also produced to test for the sensitivity of the assay to detect mixed infections.

### Statistical analysis

One-way ANOVA test using SPSS statistics software version 22 (IBM, NY, USA) was also performed to examine whether or not there was significant difference in the average T_m_ values between species. Comparison of sensitivity between microscopy and qRT-PCR-HRM assay (or PlasmoNex^TM^) in the detection of *Plasmodium* spp. in clinical samples was examined with Chi-square and Cohen’s kappa coefficient tests using SPSS statistics software (IBM).

## Results

### Primer design

**Figure 1:**
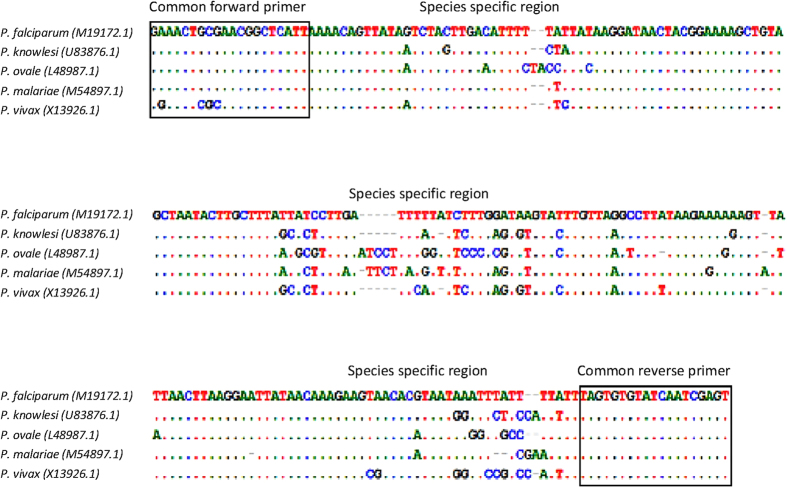
The alignment of consensus sequences of five *Plasmodium* spp. The sequences were aligned using BioEdit Sequence Alignment v7.1.7. The common forward and reverse primers flanked three species-specific regions of the five *Plasmodium* spp.

A pair of common primers was designed to amplify target sequence located on 18S SSU rRNA gene of *Plasmodium* spp.: forward primer 5′-GRAACTSSSAACGGCTCATT-3′ and reverse primer 5′-ACTCGATTGATACACACTA-3′ (Fig. 1). Seeing that the sequence of forward primer annealing site of *P. vivax* slightly varied from the other four *Plasmodium* spp., degenerate bases were incorporated into the forward primer to increase its flexibility in amplifying all the five species. According to the Primer-BLAST analysis, the PCR products might vary between 223 and 229 bp, and they covered 3 parts of species-specific regions allowing species discrimination in the melting curve analysis.

### Primer efficiency

The common primer pairs designed for qRT-PCR-HRM assay demonstrated their ability to amplify all the five *Plasmodium* spp. using conventional PCR method and gave intense bands from annealing temperatures of 59 °C to 61 °C. No unspecific product was detected in the positive control plasmids as well as the no template controls (NTCs) within the range of annealing temperatures used for PCR optimization. However, the intensity of bands for *P. falciparum*, *P. knowlesi* and *P. vivax* reduced obviously at 61 °C. As for *P. ovale*, amplifications still occurred at annealing temperatures of 62 and 63 °C. The optimal annealing temperature applied on qRT-PCR-HRM assay was finalized at 60 °C.

### qRT-PCR-HRM assay

#### Melting temperature (T_m_) for each *Plasmodium* spp

**Figure 2:**
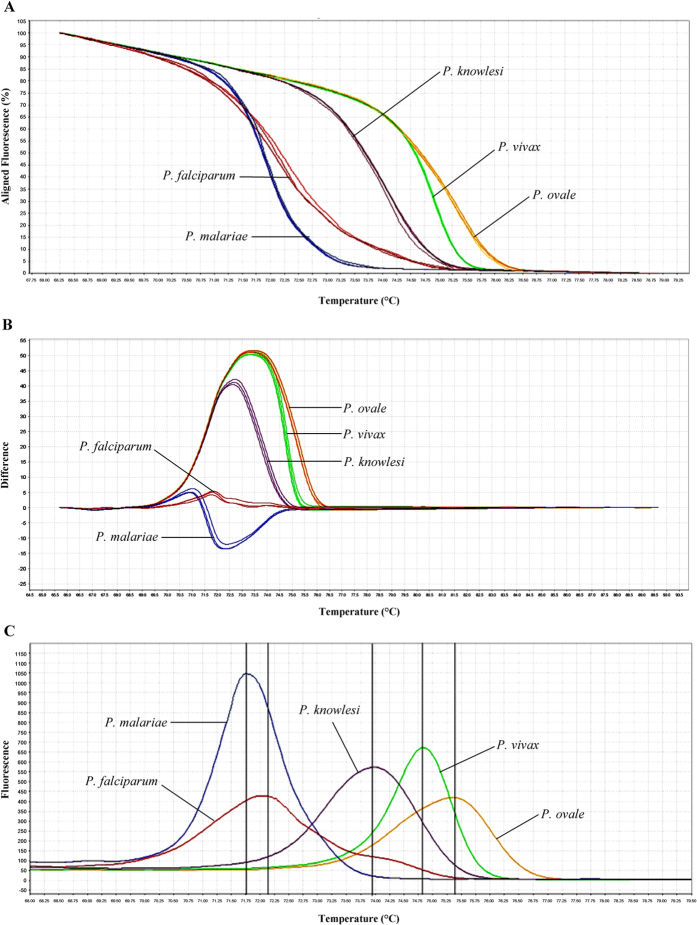
Melting curve variance of the five *Plasmodium* spp. in (A) aligned, (B) difference and (C) derivative plot analyses. The melting curve and T_m_ for each species can be very well discerned.

The qRT-PCR-HRM assay in this study was found to successfully distinguish all the five *Plasmodium* spp. The melting curve variance of the five *Plasmodium* spp. can be plotted in various forms, such as aligned and difference plots, according to the melting behaviour of their amplicons using High Resolution Melt software v 3.0.1 (Applied Biosystems) (Fig. 2). As shown in the plots, the melting curves of *Plasmodium* spp. can be distinctly separated and T_m_ for each species can be determined unambiguously. The T_m_ values were highly reproducible across 11 repeated melt curve runs.

Table 1 shows the average T_m_ with its standard deviation (SD) for each *Plasmodium* spp. identified in plasmids.

### Analytical sensitivity of the qRT-PCR-HRM assay

**Figure 3:**
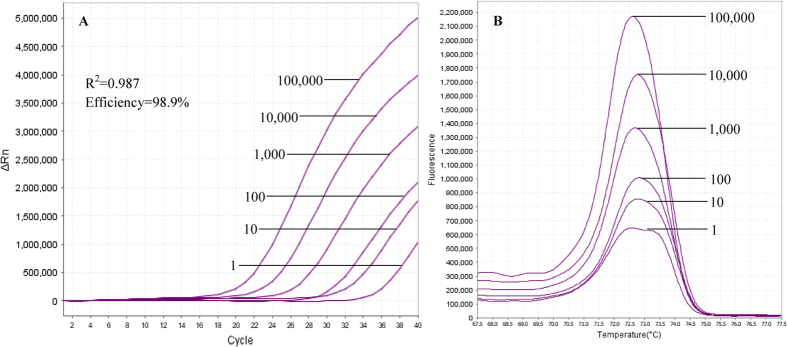
Representative amplification plot (A) and derivative melting curves (B) of 10-fold serial diluted *P. knowlesi* target sequence. Ct values of six 10-fold serial diluted plasmid DNAs were subject to standard curve analysis using 7500 Software v2.0.1 (Applied Biosystems) and the r^2^ value and assay efficiency percentage were generated as 0.987 and 98.9%, respectively. The plots also show that the LOD of the assay for *P. knowlesi* is 1 copy number of target sequence.

Ten-fold serial dilutions were done on positive control plasmid DNAs bearing target sequence [100,000 to 1 copy number(s)/μl] of each *Plasmodium* spp. to determine the sensitivity or detection limit of the qRT-PCR-HRM assay. The assay could detect target sequence of *P. knowlesi* until 1 copy number (Fig. 3). The reproducibility of these results was confirmed by duplicated tests. The lowest detectable limit of the assay for *P. vivax* was also found to be 1 copy number, whereas for *P. ovale* the limit of detection (LOD) was 10 copy numbers. Both *P. falciparum* and *P. malariae* could be detected down to 100 copy numbers of target sequence by the assay.

Standard curves plotted from the Ct’s of 10-fold serial diluted plasmid DNAs revealed that efficiency percentage achieved were 95.5%, 92.5%, 98.9%, 92.0% and 96.0% for *P. falciparum*, *P. vivax*, *P. knowlesi*, *P. malariae* and *P. ovale*, respectively (Table 2). Figure 3 shows the representative amplification plot of 10-fold serial diluted *P. knowlesi* target sequence [100,000 to 1 copy number(s)], with r^2^ value of 0.987 for its standard curve, as well as the melting curves.

### Analytical specificity of the real-time PCR coupled with HRM

**Figure 4:**
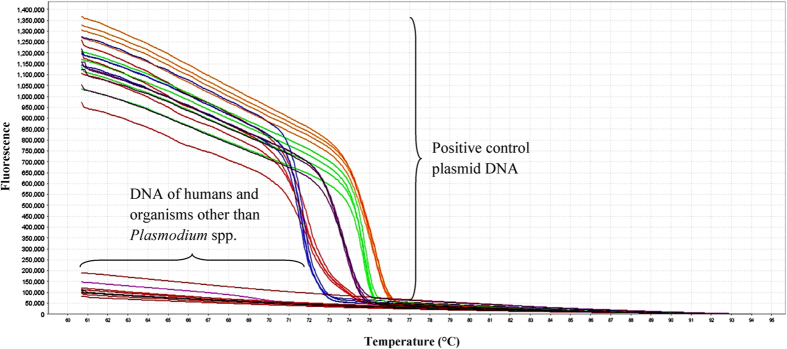
Raw data melt curve analysis of positive control plasmid DNAs and DNAs of organisms other than *Plasmodium* spp. No amplification occurred in DNA samples of non-*Plasmodium* organisms and thus no melting curve was observed.

The specificity of qRT-PCR-HRM assay was evaluated by testing DNA from other organisms (as mentioned in the methods). As seen in Fig. 4, no amplification and thus no melting curve was detected from these organisms. These results showed that this assay had high specificity to the target sequence of the five *Plasmodium* spp.

### Ability to detect mixed infections

**Figure 5:**
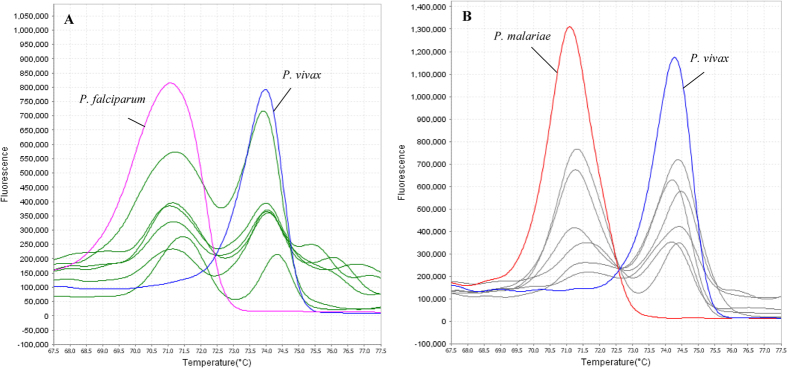
Evaluation for detection of mixed infection. Mock mixed infections with different combinations of any two *Plasmodium* spp. were created and detection ability of the assay was evaluated. The figures show the two combinations of mixed infections that could be detected by the assay, *i.e*. (**A**) *P. falciparum*/*P. vivax* and (**B**) *P. malariae*/*P. vivax*, whereby the derivative melting curve peaks corresponding to T_m_’s of each respective species can be clearly seen on the plots for each sample. Melting curves in (**A**) green and (**B**) gray were produced by samples with mock mixed infections.

Among the mock double species mixed infections, only the combinations of *P. falciparum*/*P. vivax* and *P. malariae*/*P. vivax* could be accurately distinguished by the qRT-PCR-HRM assay (Fig. 5). Two melting curve peaks contributed by the two component species could be clearly seen on the derivative melting curve plot of samples with these two combinations. However, the ratios between each species within the combinations that could give rise to this pattern were unable to be determined as the results were found to be inconsistent.

While for the other double species combinations, only one peak was observed on the derivative melting curve plot for each combination and species ratio, and the T_m_’s of the peaks were ambiguous. Thus, the assay was unsuitable to be used for detection of mixed infections other than the two above mentioned combinations.

Apart from that, the qRT-PCR-HRM assay in this study also failed to detect mixed infections with triple, quadruple and five *Plasmodium* spp. (10,000 copy numbers/species) as only one derivative melting peak with unspecific T_m_ was produced each time due to template interference.

### Screening of clinical samples

**Figure 6:**
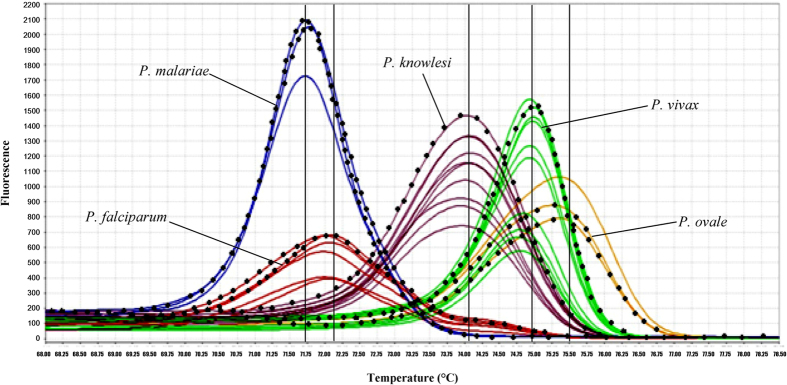
Derivative melt curve analysis for the positive control plasmid DNAs of each *Plasmodium* spp. and clinical samples. Vertical lines indicate T_m_ values for each *Plasmodium* spp. cloned in plasmids, and their melting curves are plotted with solid dotted lines while those of the clinical samples are plotted with solid colour lines. In this study, the melting curves of the clinical samples can be clustered into 5 groups and the identity of *Plasmodium* spp. present in the clinical samples can be clearly distinguished by referring their T_m_ values to those provided by the positive control plasmids.

A total of 229 clinical DNA samples collected were screened using qRT-PCR-HRM assay in this study. The identity of *Plasmodium* spp. present in all the clinical samples was initially determined using PlasmoNex^TM^. Based on that, the melting curves produced by the clinical samples were evaluated and it was observed that the *Plasmodium* spp. in the clinical samples showed consistency in their corresponding T_m_ values without much shift from those generated by positive control plasmid DNAs (Fig. 6). In the end, the melting curves resulted from the clinical samples could be clustered into five groups using auto-calling mode of High Resolution Melt software v 3.0.1 (Applied Biosystems), and the identification of the *Plasmodium* spp. present in the clinical samples could be done by referring their T_m_ values to those of plasmids. The confidence interval for auto-called results ranged between 80–100%.

**Figure 7:**
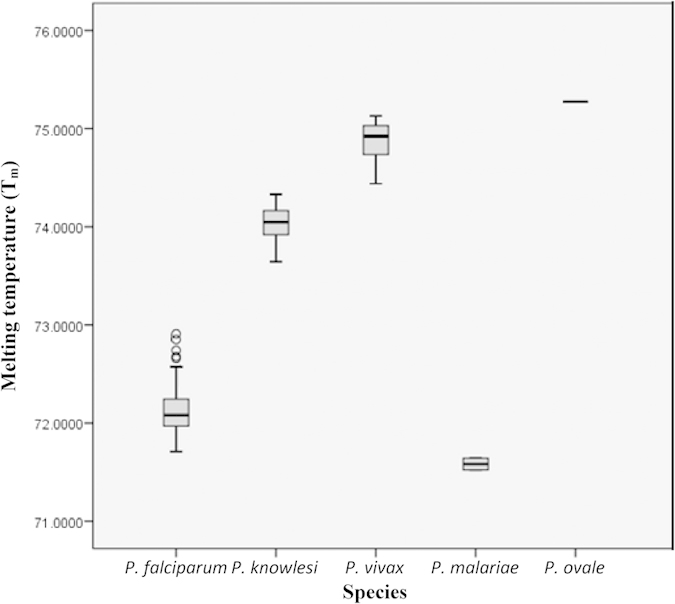
Box plot showing melting curve temperatures (T_m_) for each *Plasmodium* spp. Note that *P. malariae* and *P. ovale* had only 2 and 1 sample(s), respectively. Four outliers are noticed for *P. falciparum*. The ANOVA test showed significant difference of average T_m_ values between species (*p* = 0.001).

From the screening results, *Plasmodium* spp. identified in the clinical samples were: *P. falciparum* (n = 92), *P. ovale* (n = 1), *P. malariae* (n = 2), *P. vivax* (n = 90), *P. knowlesi* (n = 36), mixed infection of *P. falciparum* and *P. vivax* (n = 2) (Table 3). There was no amplification in 6 clinical samples from the non-malaria patients with similar symptoms to malaria. All melting curve peak T_m_ for each *Plasmodium* spp. are illustrated in box plot (Fig. 7) and the average T_m_ with SD for each species was shown in Table 1. The ANOVA test also showed that T_m_ values between species could be significantly differentiated by having *p*-value of 0.001. Overall, the results produced by the qRT-PCR-HRM assay in this study were 100% in agreement with the previous results of PlasmoNex^TM^.

Among the clinical samples, 3 of them previously identified by microscopic examination as two *P. falciparum* and one *P. vivax*-infected cases were otherwise identified as negative by both PlasmoNex^TM^ and qRT-PCR-HRM assay (Table 3). As for the 14 clinical samples from patients with presumptive clinical diagnosis of malaria but were microscopically negative for *Plasmodium* spp., only 3 of them showed negative results in qRT-PCR-HRM assay while the other 2 were identified as *P. falciparum*-infected specimens, 4 were identified as *P. knowlesi*, and the remaining 5 were *P. vivax*-infected specimens. The same results were also obtained by using PlasmoNex^TM^.

**Figure 8:**
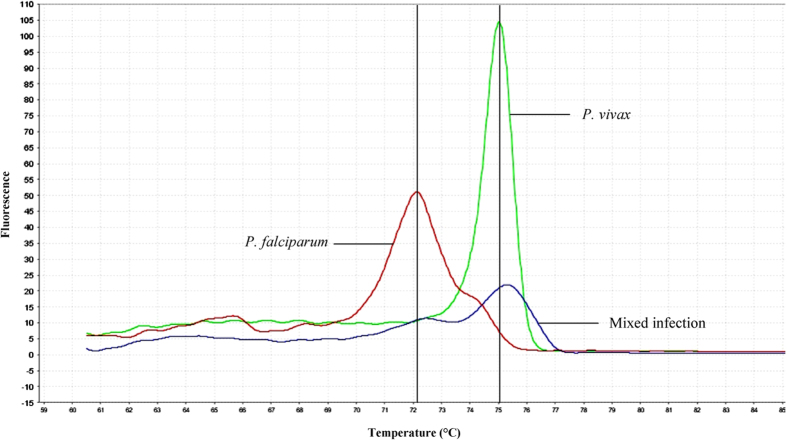
Derivative melt curve analysis for the positive control plasmids cloned with *P. falciparum* and *P. vivax*, and a clinical sample with mixed infection. The melting curve of this clinical sample shows two significant peaks with T_m_ values close to those of the two plasmid controls (vertical lines). This denotes that the clinical sample contains mixed infection of *P. falciparum* and *P. vivax*.

The other two clinical samples which were previously diagnosed by PlasmoNex^TM^ as mixed infections of *P. falciparum* and *P. vivax* were also detected as mixed infections using qRT-PCR-HRM assay in this study (Table 3). In this case, two melting curve peaks close to the corresponding T_m_ values of *P. falciparum* and *P. vivax* were observed concurrently in the derivative melt curve plot (Fig. 8).

None of the clinical sample containing *P. knowlesi* could be identified by microscopic method. Among the 36 clinical samples identified with *P. knowlesi* by PlasmoNex^TM^ and qRT-PCR-HRM assay, 28 (78%) were misdiagnosed by microscopy as *P. malariae*, 2 (6%) as *P. falciparum*, 2 (6%) as *P. vivax*, and 4 (10%) as negative (Table 3).

In addition, another 3 cases of previously microscopically identified *P. malariae* were detected as *P. falciparum* by both qRT-PCR-HRM assay and PlasmoNex^TM^, whilst 4 cases of *P. knowlesi* were mistaken for *P. falciparum* and *P. vivax* and 1 case of *P. vivax* was initially distinguished as *P. falciparum* by microscopic method (Table 3). Microscopy also failed to identify a case mix-infection with *P. falciparum* and *P. vivax* by reporting only *P. falciparum*.

Chi-square test showed that sensitivity in detecting *Plasmodium* spp. in clinical samples between microscopy and qRT-PCR-HRM assay was significantly independent between each other (*p* *<* 0.001) with Cohen’s kappa coefficient value of 0.675 (*p* *<* 0.001) (Table 3).

## Discussion

*Plasmodium* 18S SSU rRNA gene was selected in this study because it contains both highly conserved and variable regions for *Plasmodium* spp. It has multiple copies scattered throughout the *Plasmodium* genome, which provides greater sensitivity during amplification than those single-copy genes^46^,^47^. Besides that, the RNA content in *Plasmodium* spp. is 1.5 times the amount of DNA and the rRNA subunit represents 85% to 95% of the total cellular RNA. This in turn causes the rRNA probes to have greater potential than DNA probes in molecular studies of malaria^48^. Hence, positive control clones containing partial sequence of 18S SSU rRNA gene was used to permit easy access to individual species-specific controls throughout the study.

In this study, qRT-PCR-HRM assay had been successfully developed for rapid and accurate detection of five human-infecting *Plasmodium* spp. compared to the qRT-PCR green assay developed by Oddoux and colleagues which used nine primers^31^. In contrast, a pair of primers was already adequate for our assay^31^. Our assay was found to be highly specific as none other organism than the five *Plasmodium* spp. could be amplified, and no fluorescence signal was detected in NTCs as well. The average difference in T_m_ values of 0.5 to 1 °C was sufficient enough to enable discrimination between the five *Plasmodium* spp. during the melting curve analysis. The confidence interval for auto-called results of 80–100% also indicated that the species discrimination was rather convincing. Albeit the T_m_ produced by target sequence of *P. falciparum* and *P. malariae* were very near to each other, they could still be significantly distinguishable given that *p*-value obtained from T-test between T_m_’s of both species was 0.001. Furthermore, both species could also be discerned by the shape of their melting curves, especially in the difference melting curve plot, whereby the melting curve peak of *P. malariae* pointed downwards while that of *P. falciparum* was very short and pointed upwards instead (Fig. 2). *P. falciparum* also tended to display a very small shoulder peak after the main peak in the derivative melting curve plot (Fig. 2). The melting curve profiles between species in clinical samples could be easily discriminated when positive controls were included in each run of qRT-PCR-HRM.

The performance the qRT-PCR-HRM assay in the present study was evaluated and compared with PlasmoNex^TM^ which served as the primary reference standard, given its established sensitivity and specificity over microscopic examination in malaria diagnosis^26^. It achieved 100% concordance with the results obtained using PlasmoNex^TM^ in the detection of *Plasmodium* spp. in clinical samples. The lower detection limits of PlasmoNex^TM^ was reported as 0.025, 0.25, 0.027, 0.27 and 0.15 parasites/μl for *P. vivax*, *P. knowlesi*, *P. ovale*, *P. malariae* and *P. falciparum*, respectively^26^. This implies that the qRT-PCR-HRM assay could have at least the same level of diagnostic sensitivity and specificity for *Plasmodium* spp. detection compared to PlasmoNex^TM^.

The sensitivity of the qRT-PCR-HRM assay was again justified when, likewise in PlasmoNex^TM^, it was able to detect parasitemia and identify *Plasmodium* spp. in the 11 out of 14 previously microscopically negative clinical samples (Table 3). Errors in microscopy are always reported in differentiating between *P. vivax* and *P. ovale* as well as between *P. vivax* and *P. falciparum*^49^,^50^. Besides that, the morphology of *P. knowlesi* is similar to that of *P. malariae* and correct microscopic identification between the two species is often an issue^5^. The above mentioned scenarios were exactly what occurred in 28 cases of *P. knowlesi* in this study which had been misdiagnosed as *P. malariae* by microscopy, as well as the other 8 cases. In this situation, molecular detection assays, such as qRT-PCR-HRM assay and PlasmoNex^TM^, should be brought into play to overcome these shortcomings of microscopic method. Low parasitemia levels could have also brought about the misdiagnosis of these samples during microscopic examination.

However, the presence of *Plasmodium* in the three clinical samples which was previously determined by microscopic examination, failed to be detected in qRT-PCR-HRM assay. This was in agreement with the results obtained using PlasmoNex^TM^. This may be due to the extremely low parasitemia in these samples which in turn led to the failure in or inadequate plasmodial DNA extraction from the samples, given that only 200 μl of blood was extracted for DNA sample. False positive microscopic detection of malaria can also occur when poor blood film preparation creates artefacts commonly mistaken for malaria parasites, including bacteria, fungi, stain precipitation, and dirt and cell debris^50^,^51^. Hence, although the PCR-based diagnostic methods are relatively sensitive compared to microscopic method, the plasmodial DNA extraction is the key factor that dictate the successfulness and accuracy of the diagnosis.

Malaria patients may harbour multiple species of *Plasmodium*. The qRT-PCR-HRM assay in this study was able to detect mixed infections. Yet, limitations were reported when the assay could only afford to differentiate two combinations of mixed infections, *i.e. P. falciparum/P. vivax* and *P. malariae/P. vivax*, and moreover, with unknown ratios between the species. Co-infection with *P. falciparum* and *P. vivax* is believed to be a prevalent type of mixed infection, and in Thailand, over one-third of patients manifesting acute malaria with *P. falciparum* were found to be actually infected with *P. vivax* simultaneously^52^. It was suggested that *P. falciparum* tends to suppress *P. vivax* and treatment for *P. falciparum* malaria would cause the subsequent relapse of *P. vivax*^53^. Hence, the detection for *P. falciparum*/*P. vivax* mixed infection is important for proper medical management and the assay developed in this study fits the need. This combination of mixed infection also seems common in Malaysia, as observed in clinical samples in this study and reviewed by William and Menon^54^.

As it was a quantitative real-time PCR, quantitation of parasitemia level in a clinical sample was feasible. The threshold cycle (Ct) value reflects the amount of starting material subject to qRT-PCR. The quantitation of the parasites in the clinical samples can be done by performing a comparative Ct experiment, using the standard curve constructed with plasmid control to determine the quantity of target in a sample relative to that in a reference sample^38^. However, quantitation of parasites in clinical sample was not performed in this study as there was no statistical record of parasitemia level from microscopic examination as well as PlasmoNex^TM^ available for comparison. This was also one of the advantages of qRT-PCR-HRM assay against PlasmoNex^TM^.

Similar diagnosis of malaria using nested PCR coupled with qRT-HRM approach had been reported by Kipanga and colleagues^42^. Although the method had tremendous sensitivity (limit of detection of 0.236 parasites/μl) for detecting low parasitemia in patient samples, it was very time-consuming as it involved two round of PCR and one round of melting phase. This may not be very practical to use this method of diagnosis if the case is urgent, especially in the case of *P. falciparum* infection which can lead to death within 24 hours after symptoms developed^1^,^2^. Furthermore, the method could only detect three species, *i.e. P. falciparum*, *P. malariae* and *P. ovale*, as shown in the data of this study. Thus, diagnostic method developed in this study that enables detection of all five species, including *P. knowlesi* and *P. vivax*, can be applied everywhere without geographical concern of *Plasmodium* spp. distribution. These two species together with *P. falciparum* are commonly found in the Southeast Asian region. Since copy numbers of target DNA was used as measured unit in this study, the sensitivity of assay in this study could not be compared with that of assay developed by Kipanga and colleagues, where parasites/μl was used.

Besides the aforementioned study, other similar malaria diagnostic methods using qRT-PCR approaches include those developed by Fabre *et al*.^55^, Mangold *et al*.^28^ and Oddoux *et al*.^31^ , but SYBR green was employed instead. Similar to this study, method by Mangold and team also used a pair of primers to amplify *Plasmodium* spp., yet only 4 species (excluding *P. knowlesi*)^28^ were detected. However, their method only took 1 hour to complete, which is an advantage over the method developed in this study. The average T_m_ value difference across all species ranged from 1.1–4.8 °C, and similar to this study, the T_m_ values between *P. falciparum* and *P. malariae* could be as minute as 0.3 °C. Oddoux and colleagues managed to diagnose all 5 species of human plasmodium but 9 primers were used^31^. Although qRT-PCR diagnostic methods using TaqMan chemistry, which is relatively specific, sensitive and rapid, were also designed by several groups^33^,^34^,^39^, thus far only up to 4 human *Plasmodium* spp. were on the detection list and the methods were comparatively expensive compared to HRM approach. Again, since the prevalence of *P. knowlesi* is high in the Southeast Asian region, inclusion of this species for detection is essentially important when developing a malaria diagnostic assay.

qRT-PCR-HRM technique has also been employed to develop rapid detection of various clinically important microorganisms, such as nosocomial bacteria^56^, as well as those with drug-resistance, such as rifampicin-resistant *Mycobacterium tuberculosis*^57^ and erythromycin-resistant *Bordetella pertussis*^58^. Researchers opt for qRT-PCR-based methods over conventional PCR for the development of detection assay because of their high sensitivity, although this might not be always true as sensitivity relies on many factors such as primer design^59^. However, when speaking of having wider dynamic range, rapidness, needless of post-PCR or laborious processing, and rendering quantitation analysis, qRT-PCR-HRM technique is one of the methods to be considered as it has all these advantages over conventional PCR methods. Moreover, it is also relatively cost-effective than the probe-based qRT-PCR methods.

In conclusion, the qRT-PCR-HRM assay developed in this study appears to be a promising tool for simultaneous detection and discrimination of the five human-infecting *Plasmodium* spp. by using only a single pair of primers. Despite the cost of instrument, each reaction of qRT-PCR-HRM assay costs USD 1.50–4.50, depending on the DNA extraction method used. Albeit the cost is slightly higher and reaction time is longer than those of RDT (USD 0.55–1.50) and microscopy (USD 0.12–0.40)^50^, it compensates for the sensitivity and limited species (*P. falciparum*, non-*P. falciparum* and *P. vivax*) detection issues of RDT, as well as tendency of false results and requirement of skilled microscopist for microscopic method. Owing to the advantages mentioned earlier, this assay may be a practical and potential alternative for rapid and accurate diagnosis of malaria infections. Direct loading of blood samples to the assay could be one of the improvements to be done in the future to facilitate the diagnosis especially in the remote areas.

## Additional Information

**How to cite this article**: Chua, K. H. *et al*. Development of High Resolution Melting Analysis for the Diagnosis of Human Malaria. *Sci. Rep*. **5**, 15671; doi: 10.1038/srep15671 (2015).

## Figures and Tables

**Table 1:** Average melting curve peak T_m_ value for each *Plasmodium* spp. in qRT-PCR-HRM analysis.

Species	T_m_ range (°C)		Average T_m_ ± SD (°C)	*P*-value for ANOVA test
	Plasmid clones*	Clinical samples		
*P. falciparum*	71.67–72.20	71.84–72.91	72.4 ± 0.5	
*P. malariae*	71.52–72.07	71.52–71.64†	71.5 ± 0.1	
*P. knowlesi*	73.66–74.26	73.65–74.33	73.9 ± 0.4	0.001
*P. vivax*	74.65–75.10	74.44–75.13	74.7 ± 0.3	
*P. ovale*	75.09–75.47	75.27‡	75.4 ± 0.2	

^*^1 plasmid DNA sample for each species, averaged over 22 runs on separate days.

^†^2 patients infected with *P. malariae*.

^‡^1 patient infected with *P. ovale*.

**Table 2:** R^2^ values and efficiency percentages of qRT-PCR-HRM assays for *Plasmodium* spp.

Species	R^2^ value	Assay efficiency percentage
*P. falciparum*	0.988	95.5%
*P. vivax*	0.989	92.5%
*P. knowlesi*	0.987	98.9%
*P. malariae*	0.995	92.0%
*P. ovale*	0.993	96.0%

**Table 3:**
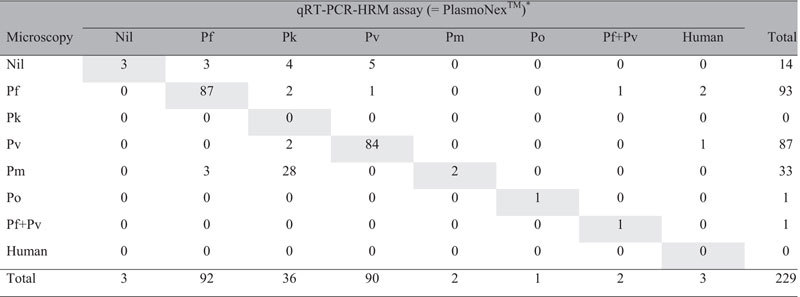
Comparison of *Plasmodium* spp. identification results for 229 clinical samples between qRT-PCR-HRM assay and PlasmoNex^TM^.

Chi-square value between microscopy and qRT-PCR-HRM assay = 756.710 (*p* < 0.001). Cohen’s kappa coefficient = 0.675 (p < 0.001). Note: Pf-*P. falciparum*; Pk-*P. knowlesi*; Pv-*P. vivax*; Pm-*P. malariae*; Po-*P. ovale*. ^*^100% concordance in results obtained from both qRT-PCR-HRM and PlasmoNex^TM^ assays. Concordant results by all three methods are shaded in grey.
